# Analysis of Residual Ridge Morphology in a Group of Edentulous Patients Seeking NHS Dental Implant Provision—A Retrospective Observational Lateral Cephalometric Study

**DOI:** 10.3390/diagnostics11122348

**Published:** 2021-12-13

**Authors:** Rafif Alshenaiber, Callum Cowan, Craig Barclay, Nikolaos Silikas

**Affiliations:** 1 Division of Dentistry, Faculty of Biology, Medicine and Health, Coupland 3 Building, University of Manchester, Manchester M13 9PL, UK; callumcowan@hotmail.com (C.C.); Nikolaos.Silikas@manchester.ac.uk (N.S.); 2 Prosthetic Dental Sciences Department, College of Dentistry, Prince Sattam Bin Abdulaziz University, AlKharj 16278, Saudi Arabia; 3 Manchester Dental Hospital, Higher Cambridge Street, Manchester M15 6FH, UK; craig.barclay@manchester.ac.uk

**Keywords:** resorption, edentulous, lateral cephalometric radiograph, residual ridge, mandible, maxilla

## Abstract

A convenience sample of 154 edentulous patients referred for implant provision at a Regional National Health Service Dental Hospital in the North West of England were identified. The cephalometric radiographs that were taken as part of the patient baseline investigation were assessed. Digital tracing was used to measure the anterior maxillary and mandibular bone height and ridge angle with respect to the maxillary and mandibular planes. The mean height of the bone in the maxilla was found to be 14 mm, and the mean ridge angle for the anterior maxillary residual ridge is 104°. The mean height of bone in the mandible was 18 mm, while the mean ridge angle for the anterior mandibular residual ridge was 77°. Using the Cawood and Howell classification demonstrated that class VI mandibles were the most common. The cross-sectional shape of the mandible varied, with the triangular shape most common. Although there was adequate bone stock for implant placement in these cases, the mandibular residual ridge resorption presents a lingual inclination to the residual bone. The limited residual ridge position and inclination would dictate that conventional implant placement could be challenging.

## 1. Introduction

The number of patients in the UK that are edentulous is falling. The Adult Dental Health Survey in 2009 revealed that 94% of adults in England, Wales and Northern Ireland retained at least one natural tooth. In the same study, it was noted that there are still about 2.7 million edentulous adults [1]. Therefore, dentists will continue to need conventional complete denture skills for the foreseeable future [2]. It is well established in the classical studies by Tallgren and Carlsson that tooth extraction results in alveolar bone resorption [3,4]. Subsequent studies have failed to agree on the rate, volume and distribution of this resorption, and there appears to be a high degree of variation between individuals. A range of factors are thought to impact the rate of resorption, such as age, gender and metabolic disorders [5]. Case-control studies contrasting denture wearers with non-denture wearers suggest that the wearing of a prosthesis itself will increase the rate of bone resorption [6,7]. If the literature were to be broadly summarised, resorption is documented to occur most rapidly in the first 3–6 months after tooth extraction with up to a 50% reduction in ridge width at 12 months [8,9]. Both horizontal and vertical ridge resorption occurs, but most studies suggest that there is a greater horizontal than vertical component [10,11]. Resorption is thought to occur at up to four times greater rate in the mandible when compared to the maxilla [12,13]. This is complicated by the fact that the receptive pattern of the maxilla results in the residual bone stock position being upward and inward, and in the mandible the bone resorbs downward and outward, often increasing any baseline skeletal discrepancies [14].

The interplay between the clinical and technical aspects of denture construction and the patient’s psychosocial state dictates that success with complete dentures can be unpredictable. This can be frustrating and disheartening for the treating clinician. Although research has not demonstrated a strong correlation between anatomic conditions, denture quality and denture success, conventional wisdom dictates that successful dentures are more difficult to achieve in patients with severely resorbed ridges [15]. The McGill and York consensus statements have clearly defined that a two-implant overdenture is the minimum standard of care for the edentulous mandible [16,17]. Due to financial constraints, this standard of care has not been achieved for most. In those complete denture wearers that do receive dental implants, the use of prosthetic componentry to attach a denture to the implant will increase its retention. Depending on the number of implants placed and prosthetic componentry used, they can also provide significant support and, hence, compensate for poor supporting structures such and thin atrophied ridges and soft tissues. In addition, implants slow or prevent further alveolar bone resorption [18]. With implant osseointegration, and stimulation of the remaining alveolar bone, the resorptive process is reduced. This is hugely advantageous, with early and simple intervention potentially preventing progressive bone loss and, therefore, the need for the significant surgical intervention that is often needed to definitively address the severely atrophic mandible.

At Manchester dental hospital, patients who are referred after failing to tolerate conventional denture construction will be assessed at a Joint Implant Clinic. If it is agreed that the conventional options for treatment have been exhausted, the patient does not smoke and they are medically fit enough to undergo surgical treatment, funding to place dental implants will be applied for through the National Commissioning Service (NHS). The final implant plan will vary depending on the needs of the patient and the bone stock available. As part of the patients’ workup for the Joint Implant Clinic, they will have a panoramic tomography and lateral cephalometric radiographs taken. These special investigations are often sufficient to determine if adequate bone height is present to place dental implants.

The aim of the study was to assess the residual bone morphology that was present in the anterior maxilla and mandible in a cohort of patients who had failed to tolerate conventional complete dentures and were seeking NHS dental implant provision.

## 2. Materials and Methods

This is a single centre retrospective descriptive observational study. The protocol was granted ethical approval by the Health Research Authority. It was given the Integrated Research Approval System number 238472 for NHS organisations in England and a University of Manchester sponsor reference number NHS001339. The principles of the “Declaration of Helsinki”, Good Clinical Practice (GCP) and the laws and regulations of the country in which this research was conducted were adhered to.

### 2.1. Patient Selection

All edentulous patients at Manchester Dental Hospital, Manchester, UK who had been treated with dental implants or had dental implant funding approved and were awaiting treatment between June 2013 and August 2017 were assessed, and a total of 743 patients were identified. Patients were excluded from the study for the following reasons: (1) No cephalometric radiograph was taken for the patient; (2) The cut or position of the cephalometric radiograph prevented complete landmark analysis; (3) There was evidence of residual dentition or dental implants on the cephalometric radiograph; (4) There was evidence of mini-screws or plates on the radiograph indicating previous trauma or bone grafting.

This resulted in a convenience sample of 154 patients. A total of 146 maxillary and 152 mandibular edentulous arches were included in the study.

**Figure 1:**
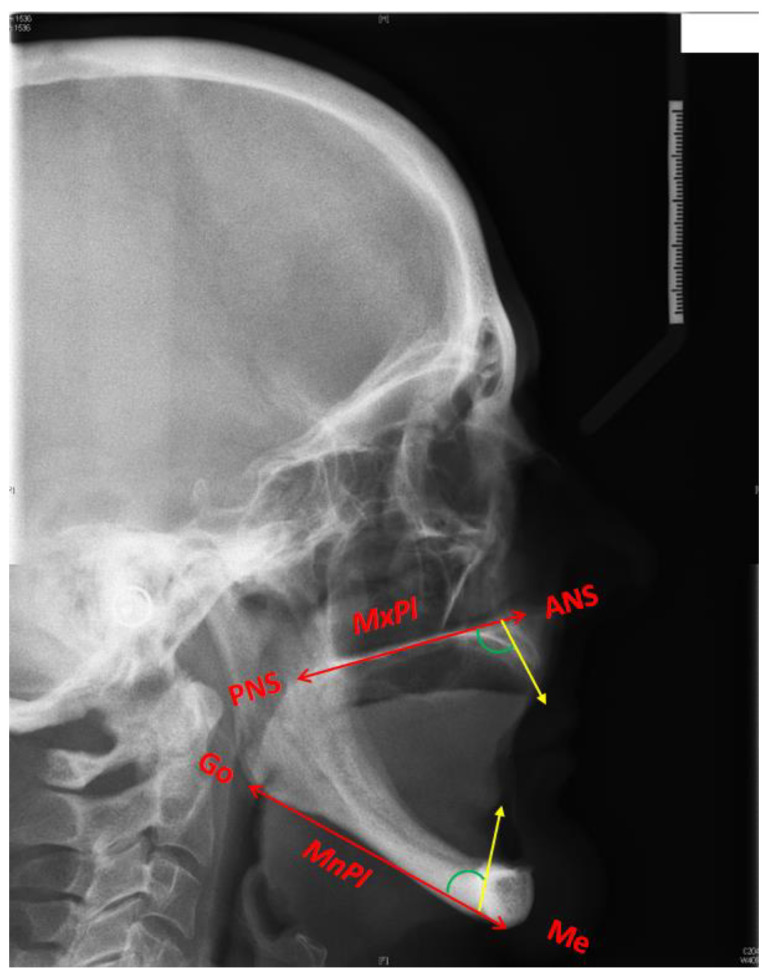
Tracing template used in this study: the red line in the maxillae connecting the anterior and posterior nasal spine (ANS-PNS line) represents the maxillary plane reference (MxPl); the red line in the mandible connecting the menton and the gonion (Me-Go line) represents the mandibular plane reference (MnPl); the yellow line in the maxillae is the line bisecting the maxillary residual ridge; the yellow line in the mandible is the line bisecting the mandibular residual ridge. The angles in green represent the maxillary and the mandibular incisal inclination adopted from dentate tracing.

Each lateral cephalometric radiograph was taken using Planmeca Promax^®^ (Planmeca UK Limited, Coventry, UK), they were anonymised before being traced by a single examiner. This was completed on the Picture Archiving and Communication System (PACS) imaging system (Insignia Medical System, Basingstocke, UK) using the annotation functions. The key anatomical landmarks were identified and, using the dentate cephalometric tracing, tracing lines were also added to the tracing presented in Table 1. An example tracing template is shown in Figure 1.

**Figure 2:**
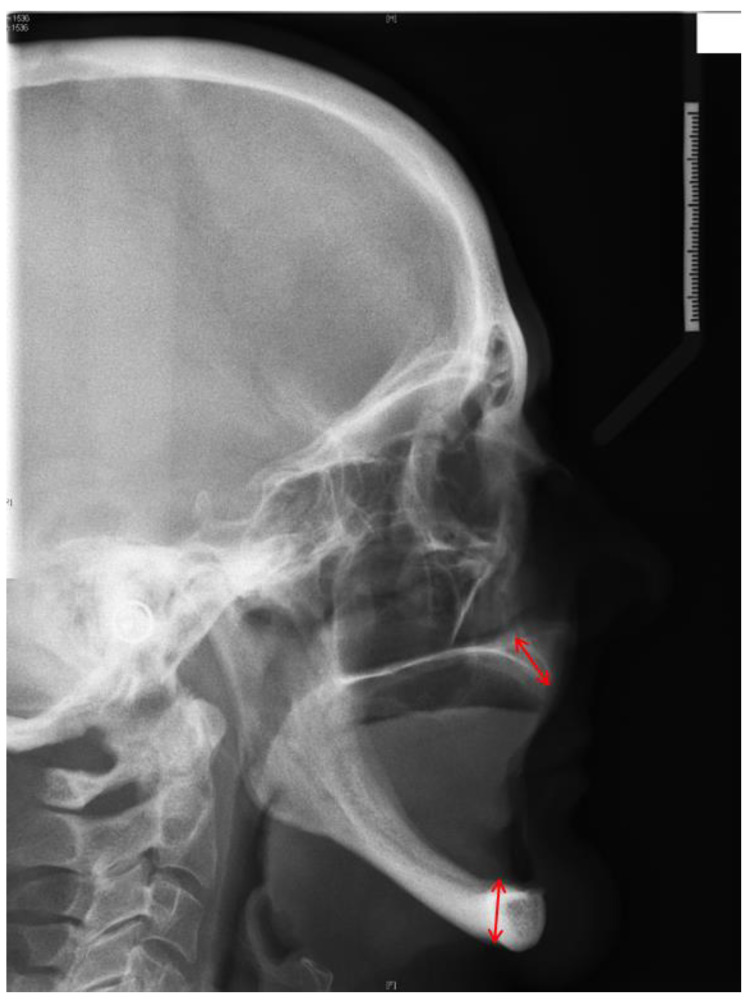
The red lines represent the height of the anterior part of maxillary (**top**) and mandibular arches (**bottom**).

A line was then drawn from the maximum midpoint of the ridge to the maxillary and mandibular plane angles. This line would transect the point on the residual alveolar ridge of maximum height (Figure 2). The difference in height between the maxillary or mandibular plane and the maximum height of the residual ridge was then measured.

### 2.2. Statistical Analysis

All data were analysed using IBM SPSS Version 22.0 (IBM Corp. Released 2013. IBM SPSS Statistics for Windows, Version 22.0 Armonk, NY, USA: IBM Corp). The data were assessed for normality using a Kolmogorov-Smirnov test and histograms. Intra-operator reliability was assessed using Cohen’s kappa correlation coefficient.

## 3. Results

These tests showed the data to be normally distributed. Summary statistics are therefore displayed as mean and standard deviation.

Intra-operator reliability was measured using a test–retest process. Each anonymised Cephalometric tracing was allocated a number. Three weeks after the initial tracings, a random number generator was used to select 30 cases from the original 154. The radiographs were then retraced and compared to the original. Results reliability was demonstrated using a correlation coefficient (Table 2).

### 3.1. Residual Alveolar Ridge Height

The mean residual alveolar ridge height of the maxilla was 14.09 mm ± 3.78 and 18.94 mm ± 5.57 for the mandible.

**Figure 3:**
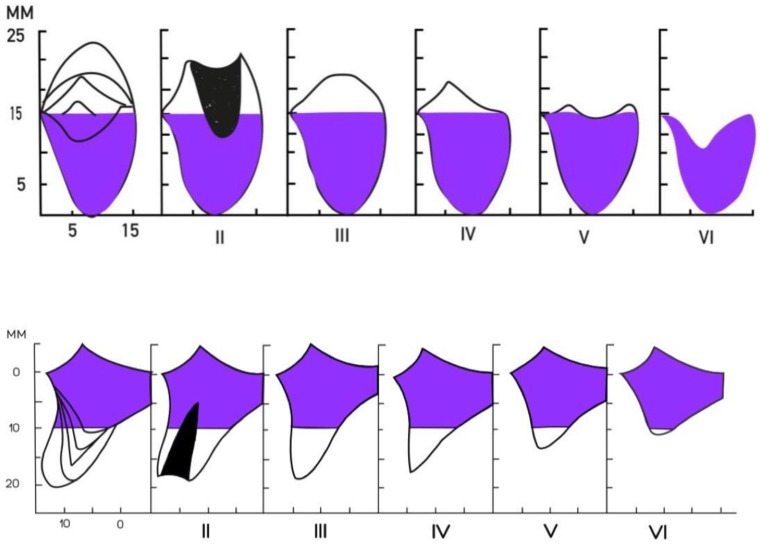
Diagrammatic summary of the Cawood and Howell Classification for the Maxilla (**above**) and the Mandible (**below**).

In order to give a more clinical context to the results, the measurements have been subclassified according to the Cawood and Howell classification (Figure 3). Table 3 shows the percentage of Cawood and Howell Subgroups in the maxilla and mandible.

### 3.2. Residual Alveolar Ridge Inclination

The mandibular residual ridge/mandibular plane angle and maxillary residual ridge/maxillary plane angle are also to be calculated (Table 4).

### 3.3. Mandibular Alveolar Bone Cross-Section

**Figure 4:**
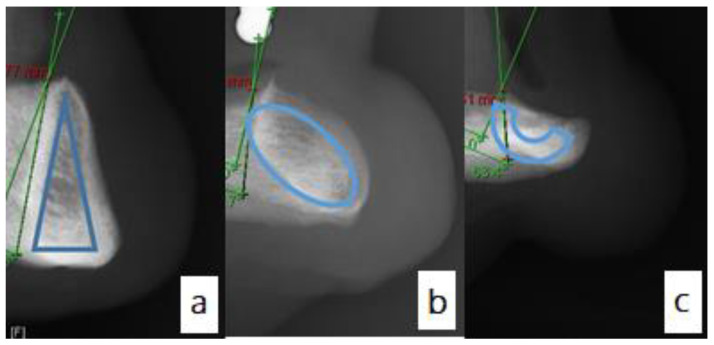
The distinctive shapes of the cross-sectional view of the anterior edentulous mandible; (**a**): triangular shape, (**b**): lingually inclined oval shape and (**c**): Inverted C shape.

Figure 4 shows the three distinct cross-sectional shapes of the anterior mandible. The majority of the mandibles assessed presented with the classic triangular cross-section. This was seen in 44.7% of cases (Figure 4a). The lingually inclined oval shape was seen in 38.2% of cases (Figure 4b). The inverted C was noted in 17.1% of cases (Figure 4c).

The shape of the mandibular ridge was also assessed within the Cawood and Howell classification subgroups (Table 5).

## 4. Discussion

This single-centre retrospective descriptive observational study explores the amount and inclination of residual bone stock that was present in the anterior maxilla and mandible in a cohort of patients seeking NHS implant-based rehabilitation after failing to tolerate conventional dentures. It must be recognised that the study design is limited and may be subject to significant selection bias. This must be considered when applying the findings to a wider cohort of denture wearers.

The failure to tolerate conventional dentures does not correlate exactly with advanced resorption. It is well recognised that some edentulous patients with high well-rounded ridges fail to tolerate conventional rehabilitation. This can be for a myriad of reasons, such as gagging or compromised support due to muco-gingival conditions. This study does not examine this; it aims only to broadly assess the variations in bone stock that you are likely to find in a typical cohort of this sort, and therefore allow conclusions to be drawn about the most common type of patients.

The data shows that the residual alveolar ridge height was 14.09 mm in the maxilla and 18.94 mm in the mandible. A lateral cephalometric radiograph is a fairly crude investigation when looking to make comment on the residual bone stock. However, their use to evaluate the residual bone is well documented in the literature [5,19,20,21,22]. Using the cephalometric study, we are able to comment on the height and shape of the residual bone in the midline. This study is limited by an inability to comment on bone width and the bone availability more posteriorly in the arch. In previous studies, this has been examined using a lateral cephalometric radiograph by undertaking sequential measurements [23,24]. However, a Cone-beam computed tomography (CBCT) would be needed to comment accurately on these parameters. In this cohort, a CBCT was not justified as part of their initial assessment.

No information is provided regarding the prosthetic plans for the patients. Overall assessment of the residual bone stock is challenging to comment on without putting it in context with a stent with radio-opaque teeth or the use of digital implant planning software with StLs or Surface Density Models of the proposed tooth position. This is a limitation of the study and provides grounds for future research and analysis. The data shows that there is great variability in the cross-sectional shape of the mandible. As the mandible becomes more resorbed, the C-shape cross-section becomes more prevalent. This is logical; with the increasing loss of the alveolar bone, the basal bone becomes more prominent, taking on a relatively predictable pattern. That said, we need to be careful not to assume this will always be the case, with a high risk of lingual perforation possible if trying to perform flapless surgery in a mandible that is significantly resorbed but with a lingual inclination to the residual bone. Such inclination was apparent as the mean inclination angle for the anterior residual mandibular ridge was at an acute angle of 77.46°.

This result was compared to the angle formed between the mandibular plane and axis of the mandibular incisors in dentate subjects adopted from the Eastman Cephalometric standards: for Caucasians skeletal class I dentate subjects, mandibular incisal inclination is 93 ± 6°. On the other hand, the mean inclination of the maxilla as measured was 104.26°, compared to the Eastman Cephalometric standards maxillary incisal inclination of 109 ± 6°. Hence, to place a straight implant, with respect to such inclinations, with the standard prosthetic envelope in the long axis of the prosthetic supra-structure, a correction angle of about 14.26° buccally and 12.54° lingually in the maxilla and the mandible may be needed, respectively.

The mean height of bone stock available in this patient group varies. The majority of mandibles in this study were a Cawood and Howell class VI. Although there was adequate bone stock for implant placement in these cases, the limited residual ridge position and inclination would dictate that conventional implant placement could be challenging. A prosthetically-driven approach that utilises guided implant placement in such cases may be critical. Considerations to use shorter implants or angled components may present more conservative treatment options. In addition, the support available from these severely atrophied ridges may well be beyond the point that a two-implant overdenture will be sufficient to adequately address a patient’s functional needs.

## Figures and Tables

**Table 1:** Landmarks added by using the lateral cephalometric dentate tracing as guidance.

Tracing Lines	Reference
A line bisecting the maxillary residual ridge crest which represent the highest midline point of the anterior maxillary ridge; this mimics taxis of the maxillary incisor (UInc) in dentate subjects.	This line is the preferable maxillary implant position.
Maxillary mid-ridge inclination which represents the angle between the maxillary plane (MxPl) and the line bisecting the maxillary residual ridge.	The maxillary incisal inclination in dentate tracing.
A line bisecting the mandibular residual ridge which represent the midline point on the anterior mandibular ridge; this mimics the axis of the mandibular incisor (LInc) in dentate subjects.	This line is the preferable mandibular implant position
Mandibular mid-ridge inclination which represents the angle between the mandibular plane (MnPl) and the line bisecting the mandibular residual ridge.	The mandibular incisal inclination in dentate tracing.

**Table 2:** The intra-examiner agreement for all landmarks and references.

Landmarks and References	Mean Kappa Value
The mean length of the anterior residual ridges of the maxilla	0.89
The mean length of the anterior residual ridges of the mandible	0.98
The mean implant angle of placement for the anterior residual ridge of the maxilla	0.88
The mean implant angle of placement for the anterior residual ridge of the mandible	0.92

**Table 3:** Percentage and number of cases in each subgroup of the Cawood and Howell Classification.

	Class II	Class III	Class IV	Class V	Class VI
**Maxilla**	14.4%	7.5%	20.5%	29.5%	28.1%
**Mandible**	5.9%	0.7%	5.9%	13.8%	73.7%

**Table 4:** The mean inclination angle of the anterior residual ridges with the mean correction angles and standard deviations.

	Mean Inclination Angle for the Anterior Residual Ridge	Standard Deviation
**Maxillary**	104.26°	7.65
**Mandibular**	77.46°	9.11

**Table 5:** The percentages of each cross-sectional shape within Cawood and Howell classification.

	Class II	Class III	Class IV	Class V	Class VI
**Triangular**	11.8%	1.5%	11.8%	23.5%	51.4%
**Lingually inclined oval shape**	1.7%	0%	1.7%	5.2%	91.4%
**Inverted C shape**	0%	0%	0%	7.7%	92.3%

## Data Availability

Data are available upon request from the corresponding author.
